# The Role of Echocardiographic Right Atrial Strain Parameters in Evaluating Atrial Fibrillation Recurrence in Patients Undergoing Atrial Fibrillation Ablation

**DOI:** 10.3390/jcm14145155

**Published:** 2025-07-21

**Authors:** Hasan Can Konte, Emir Dervis, Idris Yakut, Dursun Aras

**Affiliations:** 1 Department of Cardiology, Istanbul Medipol University, Istanbul 34815, Türkiye; emirdervis@hotmail.com (E.D.); drdaras@gmail.com (D.A.); 2 Department of Cardiology, Sincan Training and Research Hospital, Ankara 06949, Türkiye; idrislive@windowslive.com

**Keywords:** atrial fibrillation, catheter ablation, echocardiography, strain imaging, recurrence

## Abstract

**Background:** Atrial fibrillation (AF) recurrence following catheter ablation remains a significant clinical challenge despite technological advancements, with recurrence rates in the range of 20–40%. While left atrial parameters have been extensively studied as predictors of recurrence, the contribution of right atrial mechanical function has received limited attention. The hypothesis that the combined assessment of right and left atrial strain parameters may provide superior predictive value represents an important clinical question with potential implications for post-ablation risk stratification and follow-up strategies. **Methods:** This single-center, retrospective cohort study included 100 consecutive adult patients who underwent AF ablation between May 2022 and June 2024 with at least one-year follow-up. Patients were divided into two groups: those with recurrence (*n* = 13) and those without recurrence (*n* = 87). A comprehensive echocardiographic assessment, including the speckle-tracking strain analysis of both atria, was performed. **Results:** The median follow-up was 365 days [range: 150–912 days] in patients with recurrence. In the multivariable analysis, right ventricular diameter (OR: 0.74; 95% CI: 0.61–0.90; *p* = 0.001), left ventricular end-diastolic volume (OR: 1.04; 95% CI: 1.00–1.08; *p* = 0.022), and left ventricular global longitudinal strain rate (OR: 1.22; 95% CI: 1.05–1.40; *p* = 0.007) emerged as independent predictors of recurrence. **Conclusions:** The significant association of right atrial longitudinal reservoir strain with recurrence in univariable analysis, although not retained as an independent predictor in the multivariable model, suggests the importance of comprehensive cardiac assessment including right heart parameters in predicting AF recurrence.

## 1. Introduction

Atrial fibrillation (AF) is the most common sustained cardiac arrhythmia and is associated with significant morbidity and mortality. Despite advancements in catheter ablation techniques, recurrence remains a significant clinical challenge. Several clinical factors, such as older age, female sex, and persistent AF, have been linked to recurrence risk [1,2,3].

Left atrial (LA) structural and functional abnormalities, including strain impairment, have been widely studied as predictors of AF recurrence [3,4]. However, LA strain alone has shown limited predictive accuracy in some patient groups [5,6]. In contrast, the contribution of right atrial (RA) strain remains underexplored, despite its central role in atrial conduction and hemodynamics [1,4,7].

Strain imaging, which reflects myocardial deformation during the cardiac cycle, has emerged as a sensitive marker of atrial and ventricular function [5]. While the predictive value of LA strain has been demonstrated in several studies [6], its limitations highlight the need for additional echocardiographic markers to improve risk stratification. The right atrium, which is subject to different mechanical and electrophysiological dynamics than the left atrium, may provide complementary information through strain analysis. Yet, only a limited number of studies have evaluated RA strain in the context of AF recurrence after ablation [1,7,8].

Given the complex and multifactorial nature of AF recurrence, combining RA and LA strain parameters may enhance the ability to identify high-risk patients. Integrating RA strain into the routine echocardiographic assessment may improve clinical decision-making, particularly in borderline or ambiguous cases. Furthermore, exploring the additive value of RA strain alongside conventional LA metrics may offer new perspectives in atrial cardiomyopathy and fibrosis evaluation [2,3,8].

This study aimed to investigate the predictive value of right atrial strain, in combination with left atrial echocardiographic parameters, for identifying patients at risk of AF recurrence after catheter ablation. We hypothesized that incorporating RA strain would enhance the predictive accuracy of conventional LA-based models and help refine risk stratification strategies in post-ablation follow-up.

## 2. Materials and Methods

### 2.1. Study Design and Population

This single-center, retrospective, observational cohort study was conducted at Istanbul Medipol Medical School between May 2022 and June 2024. Adult patients (≥18 years) who underwent atrial fibrillation (AF) ablation with successful pulmonary vein isolation and had at least one-year follow-up were consecutively enrolled. Patients were divided into two groups based on the presence or absence of AF recurrence during the follow-up period: the recurrence group [Group Rec (+)] and the non-recurrence group [Group Rec (−)]. The study protocol was approved by the institutional ethics committee (E-10840098-202.3.02-5330. Date: 9 September 2024), and the study was conducted in accordance with the Declaration of Helsinki. Due to the retrospective design, informed consent was waived.

### 2.2. Inclusion and Exclusion Criteria

The inclusion criteria comprised: (1) adult patients aged ≥18 years; (2) diagnosed paroxysmal or persistent atrial fibrillation; (3) patients who underwent AF ablation with successful pulmonary vein isolation; and (4) at least one-year follow-up after the procedure.

The exclusion criteria were: (1) presence of valvular pathologies that could affect right atrial mechanical functions; (2) inadequate echocardiographic imaging quality; (3) mechanical prosthetic valves; (4) presence of left atrial thrombus; (5) patients deemed unsuitable for ablation procedure; (6) failure to achieve sinus rhythm after ablation either spontaneously or with electrical cardioversion; and (7) follow-up period shorter than one year.

### 2.3. Data Collection

Demographic and clinical data were retrospectively collected from hospital records and information systems. Technical procedural data were obtained from catheterization laboratory records. As a standard institutional practice, patients were scheduled for monthly outpatient follow-up visits after the procedure. All data regarding at least one-year outpatient follow-up were retrieved from the hospital information system and recorded in a standardized data collection form.

Demographic variables included age, sex, weight, height, body mass index (BMI), smoking history, and comorbidities (hypertension, diabetes mellitus, obstructive sleep apnea, chronic obstructive pulmonary disease, chronic renal failure, coronary artery disease, congestive heart failure, and thyroid disorders). Clinical variables comprised CHA_2_DS_2_-VASc score, HAS-BLED score, and vital signs (heart rate, systolic and diastolic blood pressure). AF-related data included AF history, duration (months), type of attacks (paroxysmal, persistent, or long-standing persistent), and previous catheter ablation history.

### 2.4. Echocardiographic Assessment

All patients underwent comprehensive transthoracic echocardiographic evaluation before the ablation procedure using a Vivid S70N Ultra Edition (GE Healthcare, Chicago, IL, USA). Standard views were obtained according to the recommendations of the American Society of Echocardiography. Echocardiographic parameters included right and left atrial dimensions, right and left ventricular dimensions and function, and strain parameters.

Speckle-tracking echocardiography was used to assess atrial strain parameters. Right and left atrial longitudinal reservoir strain, conduit strain, and contractile strain were measured. Strain analysis was performed using Vivid S70N Ultra Edition (GE Healthcare, Chicago, IL, USA) with the EchoPAC software version 206 on stored digital loops with adequate frame rates (≥60 frames/second). The endocardial border of the right and left atrium was manually traced, and the region of interest was adjusted to include the entire atrial wall thickness. The onset of the P wave on electrocardiography was used as the reference point for strain measurements in patients with sinus rhythm.

All echocardiographic measurements were performed by two experienced echocardiographers who were blinded to the clinical data and patient outcomes. Inter-observer variability was assessed in 20 randomly selected patients (20% of the cohort). Intra-class correlation coefficients were 0.88 (95% CI: 0.82–0.92) for right atrial longitudinal reservoir strain, 0.91 (95% CI: 0.86–0.94) for left atrial longitudinal reservoir strain, and 0.89 (95% CI: 0.84–0.93) for left ventricular global longitudinal strain rate. Intra-observer variability, evaluated in 15 patients with repeated measurements after 2 weeks, yielded ICC values exceeding 0.90 for all strain parameters.

### 2.5. Ablation Procedure and Follow-Up

Atrial fibrillation ablation procedures were performed according to the institutional protocol, which included either radiofrequency or cryoablation techniques. The technical details of the ablation procedures were recorded, including the type of catheter used and the achievement of pulmonary vein isolation.

Post-ablation, all patients were followed up with regular outpatient visits as per institutional protocol. The first three months after ablation were considered as the blanking period, and any AF episodes during this period were not classified as recurrence, in accordance with current guidelines. Recurrence was defined as any documented episode of atrial fibrillation, atrial flutter, or atrial tachycardia lasting ≥30 s after the blanking period. The diagnosis of recurrence was based on 12-lead electrocardiogram, Holter monitoring, or cardiac event recorders during the follow-up period.

For patients who experienced recurrence, additional data regarding the time to recurrence and subsequent interventions (repeat ablation, electrical cardioversion, or medication adjustments) were collected.

### 2.6. Statistical Analysis

Descriptive statistics were used to summarize the data obtained from the study. Continuous variables (such as age, body mass index, body surface area, heart rate, blood pressure values, and echocardiographic measurements) were presented as median [minimum–maximum] due to their non-normal distribution, as indicated by normality tests. These variables are marked with “§” in the tables. Categorical variables (such as sex, smoking status, presence of comorbidities, atrial fibrillation types, and medication use) were summarized as counts and percentages *n* (%) and are marked with “‡” in the tables.

The normality of the data was assessed using the Shapiro–Wilk test (for *n* < 50) and visual methods (histogram and Q-Q plots).

Comparisons between groups with recurrence *(n* = 13) and without recurrence (*n* = 87) were performed using Pearson’s chi-squared test for categorical variables in 2 × 2 tables where the expected cell count was ≥5 and Fisher’s Exact Test for 2 × 2 tables with expected cell counts < 5. The Fisher–Freeman–Halton test was used for R × C tables. These tests were marked with “*” in the tables. The Mann–Whitney U test was applied for continuous variables due to non-normal distribution, marked with “**” in the tables.

To predict recurrence, Firth’s logistic regression analysis was employed using the R software (version 4.5.0). This method was selected to mitigate separation issues and reduce estimation bias, particularly relevant given the limited number of recurrence events *(n* = 13). The analysis was conducted utilizing the logistf, pROC, dplyr, and car packages. Multicollinearity among predictors was evaluated using the Variance Inflation Factor (VIF), and all included variables demonstrated VIF values below 1.2, indicating no significant multicollinearity concerns.

Model performance was assessed through several metrics, including the area under the receiver operating characteristic curve (AUC), the Hosmer–Lemeshow goodness-of-fit test, sensitivity, specificity, overall accuracy, and 5-fold cross-validation to ensure model stability. For variable selection, an initial univariable screening was conducted, followed by a stepwise backward elimination approach (using a threshold of *p* > 0.10) to reduce the risk of overfitting. Both statistical significance and clinical relevance were considered throughout the selection process. Model comparisons were guided by the Akaike Information Criterion (AIC) and the Bayesian Information Criterion (BIC).

During the construction of the multivariable model, four variables that were statistically significant in the univariable analysis—right ventricular diameter, left ventricular global longitudinal strain rate, left atrial diameter, and left ventricular end-diastolic volume—were initially included based on their clinical importance and established evidence in the literature. Although left ventricular end-diastolic volume was only marginally significant in univariable analysis (*p* = 0.082), it remained in the final model following the exclusion of left atrial diameter (which exceeded the *p* > 0.10 threshold during backward elimination).

Other strain-related parameters—right atrial longitudinal reservoir strain, tricuspid annular plane systolic excursion, and left atrial longitudinal reservoir strain—were also significant in univariable analysis. However, due to the limited number of recurrence events (*n* = 13), these variables were excluded from the final model to preserve model parsimony and prevent overfitting.

To examine the prognostic value of various echocardiographic parameters in predicting recurrence after atrial fibrillation ablation, a comprehensive analytical methodology was implemented. Four distinct predictive model categories (A-D) were constructed to determine the incremental value of atrial and ventricular parameters:

- Model A: All Atrial Parameters (right atrial strain + left atrial strain + left atrial diameter).
- Model B: Right Heart Parameters (right atrial strain + right ventricular diameter + tricuspid annular plane systolic excursion).
- Model C: Left Heart Parameters (left atrial strain + left atrial diameter + left ventricular strain rate).
- Model D: All Strain Parameters (right atrial strain + left atrial strain + left ventricular strain rate).

In each model category, a stepwise approach was adopted from baseline univariable models (Model 1) to bivariable (Model 2) and trivariable (Model 3) models. The specific parameters included in each model were as follows:

In Model A (All Atrial Parameters):

- Model 1 included Right Atrial Longitudinal Reservoir Strain.
- Model 2 added Left Atrial Longitudinal Reservoir Strain.
- Model 3 incorporated Left Atrial Diameter as a third parameter.

In Model B (Right Heart Parameters):

- Model 1 included Right Atrial Longitudinal Reservoir Strain.
- Model 2 added Right Ventricular Diameter.
- Model 3 incorporated Tricuspid Annular Plane Systolic Excursion.

In Model C (Left Heart Parameters):

- Model 1 included Left Atrial Longitudinal Reservoir Strain.
- Model 2 added Left Atrial Diameter.
- Model 3 incorporated Left Ventricular Global Longitudinal Strain Rate.

In Model D (All Strain Parameters):

- Model 1 included Right Atrial Longitudinal Reservoir Strain.
- Model 2 added Left Atrial Longitudinal Reservoir Strain.
- Model 3 incorporated Left Ventricular Global Longitudinal Strain Rate.

The discriminative power of each model was evaluated using ROC (Receiver Operating Characteristic) curve analysis, and the area under the curve (AUC) was calculated. To compare model performances, *p*-values for differences in AUC values were calculated in comparison to the reference model (Model 1). To enhance the reliability of prognostic assessment, Integrated Discrimination Improvement (IDI) and continuous Net Reclassification Index (NRI) parameters were also calculated to quantify the prediction accuracy of the models.

A post hoc power analysis was conducted to evaluate the adequacy of our sample size. Given the limited number of recurrence events (*n* = 13), we specifically employed Firth’s penalized maximum likelihood estimation to address small-sample bias. Power calculations revealed that univariable analyses achieved 66–85% power for individual predictors: 76% for right atrial strain (OR = 0.92), 85% for right ventricular diameter (OR = 0.74), 71% for left atrial strain (OR = 0.91), and 66% for TAPSE (OR = 0.84). The multivariable model with three predictors (events-per-variable ratio = 4.3:1) yielded approximately 46% power. Despite this limitation, Firth’s method ensures more reliable estimates than standard logistic regression in this context. Model stability was confirmed through 5-fold cross-validation (AUC = 0.830) and 1000 bootstrap resamples (bootstrap-corrected AUC = 0.825).

Statistical analyses were conducted using Jamovi (version 2.3.28) and JASP (version 0.19.2). A *p*-value ≤ 0.05 was considered statistically significant.

## 3. Results

A total of 100 patients were included in the study, with 13 (13.0%) experiencing recurrence [Group Rec (+)] and 87 (87.0%) without recurrence [Group Rec (−)].

In Group Rec (+), the median time to recurrence was 365 days (range: 150–912 days). The median age of the study population was 60.5 years (range: 19.0–79.0), with a higher median age in Group Rec (+) compared to Group Rec (−), though this difference was not statistically significant (67.0 vs. 59.0 years, *p* = 0.164). Female patients were more prevalent in Group Rec (+) (69.2%) than in Group Rec (−) (39.1%), but the difference was not statistically significant (*p* = 0.080).

The median CHA_2_DS_2_-VASc score was significantly higher in Group Rec (+) than in Group Rec (−) (*p* = 0.012), whereas the proportion of patients with CHA_2_DS_2_-VASc scores ≥2 was similar between the groups (*p* = 0.360). Other demographic and clinical characteristics are summarized in Table 1.

A history of atrial fibrillation was present in 92% of the study population, with no significant difference between groups (*p* = 0.592). Details of atrial fibrillation history and treatment characteristics are presented in Table 2. However, the distribution of atrial fibrillation types varied significantly (*p* = 0.013). The proportion of patients with paroxysmal atrial fibrillation was lower in Group Rec (+) (23.1% vs. 60.9%), while long-standing persistent atrial fibrillation was more frequent (38.5% vs. 10.3%) compared to Group Rec (−).

The distribution of recurrence events across AF subtypes (paroxysmal *n* = 3, persistent *n* = 5, long-standing persistent *n* = 5) precluded robust statistical comparisons, and these findings should be considered descriptive.

Although early recurrence was more common in Group Rec (−) (10.3%) than in Group Rec (+) (7.7%), the difference was not statistically significant (*p* = 0.999).

Hemodynamic and echocardiographic findings are presented in Table 3. Median systolic blood pressure and heart rate were comparable between groups (*p* = 0.051 and *p* = 0.735, respectively), but diastolic blood pressure was significantly lower in Group Rec (+) (*p* = 0.040). Among echocardiographic parameters, right atrial longitudinal reservoir strain was significantly lower in Group Rec (+) than in Group Rec (−) (*p* = 0.049). Additionally, right ventricular and left atrial diameters were significantly larger in Group Rec (+) (*p* = 0.044 and *p* = 0.020, respectively). Other echocardiographic parameters did not differ significantly between groups (*p* > 0.05) (Table 3).

Among patients with recurrence, electrical cardioversion was performed in nine (69.2%), while radiofrequency ablation and other ablation procedures were performed in 23.1% and 7.7% of cases, respectively.

Firth’s logistic regression analysis was utilized to identify predictors of recurrence (Table 4). In the univariable analysis, tricuspid annular plane systolic excursion, right atrial longitudinal reservoir strain, right ventricular diameter, left atrial longitudinal reservoir strain, and left atrial diameter were all significantly associated with recurrence (*p* < 0.05). An increase in the left ventricular global longitudinal strain rate was found to markedly elevate the risk of recurrence (OR: 1.22; 95% CI: 1.06–1.42; *p* = 0.007).

In the multivariable model, only three variables emerged as independent predictors: right ventricular diameter (OR: 0.74; 95% CI: 0.61–0.90; *p* = 0.001), left ventricular end-diastolic volume (OR: 1.04; 95% CI: 1.00–1.08; *p* = 0.022), and left ventricular global longitudinal strain rate (OR: 1.22; 95% CI: 1.05–1.40; *p* = 0.007). Although left ventricular end-diastolic volume did not reach conventional statistical significance in the univariable analysis (*p* = 0.082), it emerged as a statistically significant predictor within the multivariable framework.

The final model demonstrated strong discriminatory power, with an area under the ROC curve (AUC) of 0.844, indicating robust performance in distinguishing patients with and without recurrence. Model performance metrics revealed high sensitivity (98.9%) but limited specificity (30.8%), yielding a positive predictive value of 16% and negative predictive value of 97%. These characteristics indicate that patients predicted as low-risk have a 97% probability of remaining recurrence-free, while among those classified as high risk, only 16% will experience recurrence during follow-up. The post hoc power analysis demonstrated that, while individual predictors achieved 66–85% power, the multivariable model was limited to 46% power due to the constrained events-per-variable ratio.

In our predictive model analysis examining the prognostic value of various echocardiographic parameters in predicting recurrence after atrial fibrillation ablation (Table 5), Model 3 of Model B (Right Heart Parameters), which includes the combination of Right Atrial Longitudinal Reservoir Strain, Right Ventricular Diameter, and Tricuspid Annular Plane Systolic Excursion parameters, exhibited the highest discriminative capability (AUC = 0.794, 95% CI: 0.678–0.910). However, this improvement did not reach statistical significance compared to the baseline model (*p* = 0.244). Among all evaluated models, the only statistically significant improvement was observed in the continuous NRI value in Model 3 of Model C (Left Heart Parameters) (NRI = 0.741, 95% CI: 0.202–1.280, *p* = 0.007). This model incorporates Left Atrial Longitudinal Reservoir Strain, Left Atrial Diameter, and Left Ventricular Global Longitudinal Strain Rate parameters.

When comparing across model categories, higher AUC values were achieved in models that included ventricular parameters (Models B and C) compared to those utilizing atrial strain parameters alone (Models A and D). This finding suggests that the combination of atrial and ventricular parameters may provide a stronger predictive performance in forecasting atrial fibrillation recurrence.

## 4. Discussion

The present study demonstrates the role of RASr-measured 2D-STE after AF ablation in predicting AF recurrence in patients with AF ablation.

The initiation of AF is generally sustained by rapidly discharging drivers [9]. The pulmonary veins have been identified as the leading AF trigger site [10], and their isolation is associated with higher rates of AF freedom in patients with both paroxysmal and persistent AF [11]. This fibrotic remodeling involves the LA and the RA, as demonstrated by late gadolinium enhancement cardiac magnetic resonance [8], which identified a similar amount of LA and RA fibrosis in AF patients undergoing pulmonary vein isolation. In recent years, using the 2D-STE of the LA has proven to be a reliable surrogate of atrial fibrosis by showing a good correlation with histology [12,13] and regional late gadolinium enhancement findings [14]. The univariable significance of right atrial longitudinal reservoir strain (OR = 0.92, 95% CI: 0.86–0.98, *p* = 0.012) with 76% statistical power suggests a potentially meaningful association with recurrence. However, its loss of significance in the multivariable analysis likely reflects the limited power (46%) of our multivariable model rather than absence of a true effect. This interpretation is supported by the significant difference in median values between groups (14.0% vs. 21.0%, *p* = 0.049) and warrants evaluation in adequately powered studies before dismissing its prognostic value.

The prognostic role of left atrial functions is well established. A review reported that left atrial (LA) fibrosis is a possible significant factor and predictor in AF treatment [15]. In the DECAAF study, Marrouche et al. showed that extensive atrial tissue fibrosis identified by delayed enhancement magnetic resonance imaging (MRI) is associated with poor outcomes of AF catheter ablation [16]. Another MRI study revealed that, in patients with advanced atrial fibrosis, AF ablation is associated with a high procedural failure rate [17]. Hopman et al. concluded that bi-atrial fibrotic remodeling is present in patients with AF and also that the amount of LA fibrosis is strongly correlated with the amount of RA fibrosis [8].

In a large multiethnic population, higher RA volume indices were independently associated with incident AF [18]. Studies are showing that both LA and RA remodeling are equally associated with AF recurrence after catheter ablation and that RA remodeling may be a clinical predictor after PVI regardless of the AF type [19,20].

Our study evaluated the role of various echocardiographic parameters, particularly right atrial strain measurements, in predicting recurrence after atrial fibrillation ablation. Our findings demonstrate that right ventricular diameter, left ventricular end-diastolic volume, and left ventricular global longitudinal strain rate are strong independent predictors of recurrence. Notably, the model incorporating right heart parameters exhibited a high predictive power (AUC = 0.794), and the model including left heart parameters provided statistically significant risk reclassification (NRI = 0.741), establishing that the combined assessment of atrial and ventricular parameters offers stronger predictive performance compared to atrial strain parameters alone. Although the CHA_2_DS_2_-VASc score was significantly higher in the recurrence group (median 4.0 vs. 2.0, *p* = 0.012), we did not incorporate it into our multivariable model for methodological and conceptual reasons. First, our primary objective was to evaluate echocardiographic strain parameters as direct mechanical correlates of recurrence. Second, including this composite score would have reduced our events-per-variable ratio to 3.25:1, substantially increasing overfitting risk. Third, individual components of the score (age, sex, hypertension, diabetes, heart failure) were already represented in our dataset, potentially introducing multicollinearity. Future studies with at least 40–50 recurrence events should explore integrated models combining clinical risk scores with echocardiographic parameters.

Current challenges in predicting atrial fibrillation (AF) recurrence have prompted clinicians to search for more reliable predictive models. Our study demonstrates that evaluating right atrial mechanical functions integrated with left atrial parameters may provide a robust approach to predicting AF recurrence. Tomaselli et al. [1] investigated the value of right atrial strain analysis in predicting AF recurrence after electrical cardioversion and found that right atrial reservoir strain significantly correlated with recurrence risk. Similarly, in our study, right atrial longitudinal reservoir strain showed a significant association with recurrence in univariable analysis (OR: 0.92, 95% CI: 0.86–0.98). This finding supports the importance of right atrial mechanical functions in the pathophysiology of AF and recurrence risk.

The clinical value of atrial strain parameters has become the focus of increasing research in recent years. A systematic review by Barilli et al. [6] highlighted the potential role of left atrial strain in predicting AF recurrence after catheter ablation therapy. Our study extends these findings by demonstrating that right atrial strain parameters carry significant predictive value. Particularly noteworthy is that combined models incorporating right and left atrial strain measurements (Models B and C) exhibited higher AUC values compared to models using atrial strain parameters alone (Models A and D). This finding supports that atrial fibrillation is a complex arrhythmia developing through a bi-atrial substrate rather than a single focal point. Kiliszek et al. reported that LA strain and LAAV (LA appendage emptying velocity) can predict recurrence after AF ablation [21]. Moreno-Ruiz et al. found that left atrial strain parameters predicted AF after electrical cardioversion [22]. At the same time, Nielsen et al. reported that AF might predict atrial tachyarrhythmias after catheter ablation [23]. In another study, Moon et al. showed that right atrial anatomical remodeling was associated with early recurrence after RF AF ablation [24]. A study examining the development of AF in 142 post-CABG patients reported that right atrial dysfunction may predict the development of AF [25]. Right atrial booster strain function was predictive of SR maintenance for up to 1 year in patients with paroxysmal AF [26]. Recent research using cardiac magnetic resonance in patients with paroxysmal or persistent atrial fibrillation who underwent pulmonary vein isolation found that the right atrium experienced a progressive remodeling process, transitioning from healthy individuals to those with persistent atrial fibrillation marked by enlargement and deformation [27]. The study by Lu et al. [2] also emphasized the relationship between bi-atrial substrate and recurrence in patients with persistent AF, presenting results parallel to our findings.

Our study found that right ventricular diameter was also a strong independent predictor of recurrence (OR: 0.74, 95% CI: 0.61–0.90). This finding is consistent with the study by Hopman et al. [8] examining the role of right heart functions in the success of AF ablation. Chatterjee et al. [8,28] emphasized that the clinical use of RV morphology can be used in AF risk prediction. Right ventricular enlargement may contribute to abnormal atrial electrophysiological properties and mechanical remodeling, thereby increasing AF recurrence. Additionally, the emergence of left ventricular end-diastolic volume as a significant predictor in the multivariable model (OR: 1.04, 95% CI: 1.00–1.08) emphasizes the importance of ventriculoatrial interaction in AF recurrence.

The significantly higher CHA_2_DS_2_-VASc score in the recurrence group highlights the importance of clinical risk factors in assessing AF recurrence risk. Soysal et al. [3] emphasized the importance of the integrated evaluation of clinical, biomarker, and echocardiographic parameters in their study examining recurrence factors in cryoballoon AF ablation. Vitali et al. reported that the CHA2DS2-VASc score predicts the early recurrence of AF in the first 30 days after electrical or pharmacological cardioversion [29]. Our findings support that this integrated approach may be important in clinical practice.

In our study, the distribution of AF types showed significant differences between groups. The lower rate of paroxysmal AF (23.1% vs. 60.9%) and a higher rate of long-standing persistent AF (38.5% vs. 10.3%) in the recurrence group supports that AF duration and type are important factors affecting recurrence risk. This finding is consistent with other studies in the literature and stands out as a factor that should be considered in patient selection before AF ablation.

Among the predictive models we created, Model 3 of Model B, which includes right heart parameters (Right Atrial Longitudinal Reservoir Strain, Right Ventricular Diameter, and Tricuspid Annular Plane Systolic Excursion parameters), exhibited the highest discriminative power (AUC = 0.794, 95% CI: 0.678–0.910). This finding emphasizes the importance of using the right heart parameters to predict AF recurrence. On the other hand, Model 3 of Model C, which includes left heart parameters (Left Atrial Longitudinal Reservoir Strain, Left Atrial Diameter, and Left Ventricular Global Longitudinal Strain Rate), provided a statistically significant risk reclassification (NRI = 0.741, 95% CI: 0.202–1.280). These results demonstrate that right and left heart parameters complement the prediction of AF recurrence.

In the study by Beyls et al. [4], the value of left and right atrial strain analysis in predicting new-onset AF in patients with septic shock was investigated, emphasizing the predictive value of both atrial strain measurements. Our study, with a similar approach, supports combining the use of right and left atrial parameters in predicting AF recurrence. These findings may provide clinicians a new perspective on risk stratification and determining follow-up strategies after AF ablation.

The review by Smiseth et al. [5] on myocardial strain imaging emphasized the importance of strain parameters in evaluating cardiac mechanical functions. Our findings shed light on the potential clinical applications of strain analysis in predicting AF recurrence. In particular, the finding that an increase in left ventricular global longitudinal strain rate significantly elevates recurrence risk (OR: 1.22; 95% CI: 1.06–1.42) demonstrates the effect of ventricular functions on the AF substrate.

Naji et al. [7] reported that the left atrial volume index predicts AF recurrence only in obese patients. In our study, the lack of significant difference in body mass index between groups with and without recurrence suggests that atrial functions play an important role in AF recurrence independent of anthropometric measurements. This finding supports that atrial and ventricular functions may be more reliable for predicting AF recurrence.

### Strengths and Limitations of the Study

Our study’s strengths include comprehensive echocardiographic evaluation, appropriate statistical methodology, and comparative analysis of various predictive models. In particular, Firth’s logistic regression analysis was an appropriate approach to reducing separation issues and estimation bias in our study with a limited number of recurrence events (*n* = 13). Additionally, the various metrics used to evaluate model performance, such as ROC curve analysis, Hosmer–Lemeshow goodness-of-fit test, sensitivity, specificity, overall accuracy, and 5-fold cross-validation, increase the reliability of our findings.

Second, the limited number of recurrence events (*n* = 13) constrained our statistical power and modeling capacity. The post hoc analysis revealed 66–85% power for detecting univariable associations but only 46% power for the multivariable model. The events-per-variable ratio of 4.3:1 fell substantially below the recommended 10:1 threshold, potentially explaining why some univariably significant predictors lost significance in multivariable analysis. Although we employed Firth’s penalized regression to mitigate small-sample bias and validated model stability through cross-validation and bootstrapping, these constraints limit definitive conclusions. Based on our effect sizes, approximately 300–350 patients with 40–50 recurrence events would be required to achieve 80% power for future validation studies.

Third, while our model achieved excellent sensitivity (98.9%) for identifying patients without recurrence, the low specificity (30.8%) and positive predictive value (16%) limit its standalone diagnostic utility. The high negative predictive value (97%) suggests the model may be most useful for identifying low-risk patients suitable for standard follow-up, while those classified as high-risk would require additional risk stratification methods.

Although we employed Firth’s penalized regression—which is specifically designed to handle small-sample bias and separation issues—to mitigate these limitations and validated model stability through cross-validation and bootstrapping, these constraints limit definitive conclusions.

Moreover, our study has a retrospective design and encompasses a single-center population, which may limit the generalizability of the results. Differences in ablation techniques and recurrence assessment methods may have affected the results. In future studies, preference for a multi-center and prospective design may help overcome these limitations. In addition, a longer follow-up is required to detect late recurrence cases.

Furthermore, speckle-tracking echocardiography has limitations, including anterior chest wall deformity, difficulty in achieving an adequate frame rate, and the need to obtain optimal image quality.

Finally, our study did not evaluate other important factors that may affect AF recurrence, such as atrial fibrosis level and electrophysiological parameters. The assessment of these factors and echocardiographic parameters may provide a more comprehensive approach to predicting AF recurrence. Due to the retrospective design, advanced imaging modalities such as cardiac MRI with late gadolinium enhancement were not performed. While left atrial strain has been proposed in the literature as a non-invasive marker of fibrosis, there is limited comparable evidence for right atrial strain. Thus, the interpretation of reduced RA strain as an indicator of underlying structural remodeling or fibrosis should be made with caution. Prospective studies with integrated imaging protocols are needed to validate the relationship between RA strain and atrial fibrosis.

## 5. Conclusions

Our study emphasizes the importance of evaluating right atrial strain parameters together with left atrial and ventricular parameters in predicting recurrence after atrial fibrillation ablation. The identification of right ventricular diameter, left ventricular end-diastolic volume, and left ventricular global longitudinal strain rate as strong independent predictors of recurrence demonstrates the necessity of a comprehensive cardiac evaluation in predicting AF recurrence.

These findings may add a new dimension to the use of echocardiographic parameters in the risk stratification of patients planned for AF ablation in clinical practice. In particular, the closer follow-up of patients with long-standing persistent AF and abnormalities in echocardiographic parameters may contribute to the early determination of recurrence risk and development of appropriate treatment strategies.

In future studies, the evaluation of the relationship between right and left atrial strain parameters with electrophysiological data and atrial fibrosis measurements in larger patient populations may enable the development of more comprehensive models for predicting AF recurrence. Additionally, the investigation of the role of echocardiographic parameters in determining ablation strategies and the effectiveness of alternative treatment approaches in patients with a high recurrence risk may provide important contributions to AF management.

## Figures and Tables

**Table 1:** Demographic and clinical characteristics of patients with and without recurrence after atrial fibrillation ablation.

	Overall (*n* = 100)	Groups		
		Group Rec (+) (*n* = 13)	Group Rec (−) *(n* = 87)	*p*
Age (years) ^§^	60.5 [19.0–79.0]	67.0 [28.0–74.0]	59.0 [19.0–79.0]	0.164 **
Sex ^‡^				
Female	43 (43.0)	9 (69.2)	34 (39.1)	0.080 *
Male	57 (57.0)	4 (30.8)	53 (60.9)	
Body mass index (kg/m^2^) ^§^	29.1 [21.1–44.6]	31.2 [21.1–38.6]	29.0 [21.9–44.6]	0.392 **
Body surface area (m^2^) ^§^	1.9 [1.6–2.5]	1.9 [1.6–2.1]	1.9 [1.6–2.5]	0.502 **
Smoking ^‡^				
Non-smoker	50 (50.0)	9 (69.2)	41 (47.1)	0.437 *
Ex-smoker	26 (26.0)	2 (15.4)	24 (27.6)	
Active smoker	24 (24.0)	2 (15.4)	22 (25.3)	
Comorbidity ^‡^	82 (82.0)	12 (92.3)	70 (80.5)	0.453 *
Hypertension	60 (60.0)	11 (84.6)	49 (56.3)	0.101 *
Coronary artery disease	30 (30.0)	6 (46.2)	24 (27.6)	0.201 *
Diabetes mellitus	19 (19.0)	4 (30.8)	15 (17.2)	0.263 *
Congestive heart failure	19 (19.0)	5 (38.5)	14 (16.1)	0.068 *
Thyroid dysfunction	15 (15.0)	1 (7.7)	14 (16.1)	0.685 *
Obstructive sleep apnea	7 (7.0)	1 (7.7)	6 (6.9)	0.999 *
Chronic obstructive pulmonary disease	3 (3.0)	0 (0.0)	3 (3.4)	0.999 *
Chronic renal failure	1 (1.0)	0 (0.0)	1 (1.1)	0.999 *
Others	4 (4.0)	0 (0.0)	4 (4.6)	0.999 *
CHA_2_DS_2_-VAS_C_ score ^§^	2.0 [0.0–6.0]	4.0 [0.0–5.0]	2.0 [0.0–6.0]	**0.012** **
Groups of CHA_2_DS_2_-VAS_C_ score ^‡^				
<2	38 (38.0)	3 (23.1)	35 (40.2)	0.360 *
≥2	62 (62.0)	10 (76.9)	52 (59.8)	
HASBLED score ^§^	1.0 [0.0–3.0]	1.0 [0.0–2.0]	1.0 [0.0–3.0]	0.489 **
Medications ^‡^				
Beta blockers	78 (78.0)	13 (100.0)	65 (74.7)	0.066 *
Direct oral anticoagulants	75 (75.0)	13 (100.0)	62 (71.3)	**0.034** *
Angiotensin receptor blockers	37 (37.0)	8 (61.5)	29 (33.3)	0.066 *
Propafenon	32 (32.0)	4 (30.8)	28 (32.2)	0.999 *
Amiodarone	28 (28.0)	5 (38.5)	23 (26.4)	0.508 *
Angiotensin-converting enzyme inhibitors	19 (19.0)	1 (7.7)	18 (20.7)	0.452 *
Spironolactone	17 (17.0)	5 (38.5)	12 (13.8)	**0.043** *
Vitamin K antagonist	6 (6.0)	0 (0.0)	6 (6.9)	0.999 *
Flecainide	1 (1.0)	0 (0.0)	1 (1.1)	0.999 *
Others	18 (18.0)	3 (23.1)	15 (17.2)	0.699 *

Footnote: ^‡^: *n* (%), ^§^: Median [Min–Max.]. CHA_2_DS_2_-VASc = Congestive heart failure, Hypertension, Age ≥ 75 years, Diabetes mellitus, Stroke, Vascular disease, Age 65–74 years, Sex category; HAS-BLED = Hypertension, Abnormal renal/liver function, Stroke, Bleeding history or predisposition, Labile INR, Elderly, Drugs/alcohol. *: Pearson’s chi-squared, Fisher’s exact/Fisher–Freeman–Halton test. **: Mann–Whitney U test. Bold *p*-values indicate statistical significance (*p* ≤ 0.05).

**Table 2:** Distribution of clinical variables regarding atrial fibrillation history and current treatment in patients with and without recurrence after ablation.

	Groups		
	Group Rec (+) (*n* = 13)	Group Rec (−) (*n* = 87)	*p*
Previous atrial fibrillation ^‡^	13 (100.0)	79 (90.8)	0.592 *
Previous cardioversion for atrial fibrillation ^‡^	5 (38.5)	19 (22.1)	0.295 *
Type of atrial fibrillation attacks ^‡^			
Paroxsymal	3 (23.1) ^a^	53 (60.9) ^b^	**0.013** *
Persistent	5 (38.5) ^a^	25 (28.7) ^a^	
Long persistent	5 (38.5) ^a^	9 (10.3) ^b^	
Duration of atrial fibrillation (month) ^§^	7.0 [1.0–84.0]	5.0 [0.0–120.0]	0.893 **
Type of catheter ablation ^‡^			
Radiofrequency	9 (69.2)	53 (60.9)	0.761 *
Crioablation	4 (30.8)	34 (39.1)	
Early recurrence after ablation ^‡^	1 (7.7)	9 (10.3)	0.999 *

Footnote: ^‡^: n (%), ^§^: Median [Min–Max.]. *: Pearson’s chi-squares, Fisher’s exact/Fisher–Freeman–Halton test. **: Mann–Whitney U test. Different superscripts (a, b) within the table indicate statistical differences between groups in each row, with no statistical difference being marked where superscripts are the same. Bold *p*-values indicate statistical significance (*p* ≤ 0.05).

**Table 3:** Hemodynamic and echocardiographic parameters in patients with and without recurrence after atrial fibrillation ablation.

	Groups		
	Group Rec (+) (*n* = 13)	Group Rec (−) (*n* = 87)	*p*
Heart rate (bpm) ^§^	86.0 [47.0–140.0]	86.0 [50.0–140.0]	0.735 *
Systolic blood pressure (mm Hg) ^§^	110.0 [100.0–180.0]	128.0 [95.0–189.0]	0.051 *
Diastolic blood pressure (mm Hg) ^§^	67.0 [52.0–107.0]	78.0 [58.0–100.0]	0.040 *
Right atrial diameter (mm) ^§^	35.0 [26.0–46.0]	34.0 [25.0–47.0]	0.438 *
Right atrial volume (mL/m^2^) ^§^	42.0 [20.0–90.0]	37.0 [22.0–66.0]	0.176 *
Right atrial longitudinal reservoir strain ^§^	14.0 [4.0–30.0]	21.0 [10.0–33.0]	0.049 *
Estimated systolic pulmonary artery pressure (mmHg) ^§^	27.0 [22.0–45.0]	27.0 [20.0–52.0]	0.873 *
Right ventricular free wall longitudinal strain ^§^	24.0 [15.0–32.0]	25.0 [15.0–36.0]	0.898 *
Tricuspid S wave rate ^§^	13.0 [9.0–19.0]	14.0 [6.0–20.0]	0.161 *
Tricuspid annular plane systolic expansion (mm) ^§^	21.0 [13.0–27.0]	22.0 [12.0–30.0]	0.077 *
Right ventricular diameter ^§^	33.0 [24.0–37.0]	28.0 [21.0–36.0]	0.044 *
Left atrial diameter (mm) ^§^	44.0 [41.0–50.0]	42.0 [33.0–55.0]	0.020 *
Left atrial volume (mL/m^2^) ^§^	60.0 [35.0–110.0]	50.0 [30.0–116.0]	0.348 *
Left atrial longitudinal reservoir strain ^§^	15.0 [5.0–38.0]	22.0 [4.0–37.0]	0.052 *
Left ventricular end-diastolic volume (mL/m^2^) ^§^	85.0 [60.0–121.0]	102.0 [64.0–150.0]	0.093 *
Left ventricular end-systolic volume (mL/m^2^) ^§^	43.0 [20.0–69.0]	40.0 [21.0–104.0]	0.930 *
Left ventricular ejection fraction ^§^	50.0 [38.0–66.0]	60.0 [25.0–67.0]	0.151 *
Left ventricular global longitudinal strain rate ^§^	16.0 [6.0–24.0]	20.0 [9.0–26.0]	0.072 *
Mitral E wave rate (cm/sn) ^§^	91.0 [65.0–110.0]	88.0 [42.0–140.0]	0.845 *
Septal E’ rate (cm/sn) ^§^	7.0 [5.0–11.0]	8.0 [4.0–12.0]	0.439 *
Lateral E’ rate (cm/sn) ^§^	10.0 [7.0–14.0]	12.0 [5.0–18.0]	0.291 *
E/e’ ^§^	10.0 [8.0–22.0]	11.0 [5.0–25.0]	0.906 *

^§^: Median [Min–Max.]. *: Mann–Whitney U test. Bold *p*-values indicate statistical significance (*p* ≤ 0.05).

**Table 4:** Results of univariable and multivariable Firth’s logistic regression analysis for predicting recurrence after atrial fibrillation ablation.

**Independent Variables**	**Univariable Model**		**Multivariable Model**	
	**OR [95% CI]**	***p*-Value**	**OR [95% CI]**	***p*-Value**
Tricuspid annular plane systolic excursion (mm)	0.84 [0.72–0.97]	0.021	—	—
Right atrial longitudinal reservoir strain	0.92 [0.86–0.98]	0.012	—	—
Right ventricular diameter	1.18 [1.04–1.35]	0.009	0.74 [0.61–0.90]	**0.001**
Left atrial longitudinal reservoir strain	0.91 [0.84–0.98]	0.015	—	—
Left atrial diameter (mm)	1.15 [1.02–1.30]	0.024	—	—
Left ventricular end-diastolic volume (mL/m^2^)	1.06 [0.99–1.13]	0.082	1.04 [1.00–1.08]	**0.022**
Left ventricular global longitudinal strain rate	1.22 [1.06–1.42]	0.007	1.22 [1.05–1.40]	**0.007**

Note: OR = Odds ratio; CI = Confidence interval. Bold *p* values indicate statistical significance (*p* < 0.05). The multivariable model was derived using backward selection method. Model performance metrics: AUC = 0.844; Hosmer–Lemeshow *p* = 0.543; Sensitivity = 0.989; Specificity = 0.308; Accuracy = 0.900; Cross-validation AUC = 0.830; Log-likelihood = −28.071; AIC = 0.050; BIC = 74.563. The confusion matrix reveals that the model accurately predicts patients with recurrence (86/87) but shows lower performance in identifying patients without recurrence (4/13).

**Table 5:** Comparison of predictive models based on different echocardiographic parameters for predicting recurrence after atrial fibrillation ablation.

Prediction Models	AUC	95% CI	*p*-Value for AUC Difference	IDI	95% CI	*p*-Value for IDI	Continuous NRI	95% CI	*p*-Value for NRI
Model A: All Atrial Parameters									
Model 1	0.670	0.479–0.862	-	-	-	-	-	-	-
Model 2	0.678	0.482–0.873	0.821	0.004	−0.010–0.018	0.584	0.111	−0.470–0.693	0.707
Model 3	0.719	0.568–0.870	0.280	0.001	−0.025–0.027	0.945	0.249	−0.331–0.830	0.400
Model B: Right Heart Parameters									
Model 1	0.670	0.479–0.862	-	-	-	-	-	-	-
Model 2	0.736	0.597–0.875	0.486	0.052	−0.026–0.130	0.192	0.518	−0.048–1.084	0.073
Model 3	0.794	0.678–0.910	0.244	0.021	−0.028–0.069	0.403	0.134	−0.447–0.716	0.650
Model C: Left Heart Parameters									
Model 1	0.668	0.482–0.854	-	-	-	-	-	-	-
Model 2	0.706	0.569–0.843	0.420	0.008	−0.018–0.035	0.529	0.272	−0.308–0.852	0.357
Model 3	0.759	0.611–0.906	0.426	0.034	−0.028–0.096	0.285	0.741	0.202–1.280	**0.007**
Model D: All Strain Parameters									
Model 1	0.670	0.479–0.862	-	-	-	-	-	-	-
Model 2	0.678	0.482–0.873	0.821	0.004	−0.010–0.018	0.584	0.111	−0.470–0.693	0.707
Model 3	0.741	0.583–0.899	0.372	0.027	−0.030–0.085	0.346	0.364	−0.214–0.942	0.217

Model Specifications and Parameters: In the evaluation of Model A (All Atrial Parameters), Model 1 included the Right Atrial Longitudinal Reservoir Strain, Model 2 incorporated the Left Atrial Longitudinal Reservoir Strain, and Model 3 added the Left Atrial Diameter as a third parameter. In Model B (Right Heart Parameters), the core variable was the Right Atrial Longitudinal Reservoir Strain; Right Ventricular Diameter was added in Model 2, and Tricuspid Annular Plane Systolic Excursion (TAPSE) was included in Model 3. Model C (Left Heart Parameters) began with the Left Atrial Longitudinal Reservoir Strain (Model 1), followed by the addition of Left Atrial Diameter (Model 2), and finally the Left Ventricular Global Longitudinal Strain Rate (Model 3). Model D (All Strain Parameters) started with the Right Atrial Longitudinal Reservoir Strain (Model 1), combined it with Left Atrial Longitudinal Reservoir Strain in Model 2, and included the Left Ventricular Global Longitudinal Strain Rate in Model 3. AUC (Area Under the Curve), CI (Confidence Interval), IDI (Integrated Discrimination Improvement), and NRI (Net Reclassification Index) values were calculated for each model, using Model 1 as the reference for all statistical comparisons. Bold values in the table indicate statistical significance (*p* ≤ 0.05).

## Data Availability

The data that support the findings of this study are available from the corresponding author upon request.

## Abbreviations

The following abbreviations are used in this manuscript:

AF	Atrial Fibrillation
AIC	Akaike Information Criterion
AUC	Area Under the Curve
BIC	Bayesian Information Criterion
BMI	Body Mass Index
BSA	Body Surface Area
CI	Confidence Interval
IDI	Integrated Discrimination Improvement
LA	Left Atrium/Atrial
LVED	Left Ventricular End-Diastolic
LVEF	Left Ventricular Ejection Fraction
LVGLS	Left Ventricular Global Longitudinal Strain
NRI	Net Reclassification Index
OR	Odds Ratio
RA	Right Atrium/Atrial
RALS	Right Atrial Longitudinal Strain
ROC	Receiver Operating Characteristic
RV	Right Ventricle/Ventricular
TAPSE	Tricuspid Annular Plane Systolic Excursion
VIF	Variance Inflation Factor
